# Editorial: Recent developments in neuroimaging in mood disorders

**DOI:** 10.3389/fpsyt.2024.1371347

**Published:** 2024-02-28

**Authors:** Keita Watanabe, Jigar Jogia, Reiji Yoshimura

**Affiliations:** ^1^ Department of Radiology, Kyoto Prefectural University of Medicine, Kyoto, Japan; ^2^ School of Psychology, University of Birmingham, Dubai, United Arab Emirates; ^3^ Department of Psychiatry, University of Occupational and Environmental Health, Kitakyushu, Japan

**Keywords:** mood disorder, major depressive disorder, neuroimaging, VBM, brain network

MRI technology began to be used in hospitals in the 1980s. MRI has not only revolutionized the diagnosis of acute cerebral infarction and cerebral hemorrhage but has also increasingly been employed to visually assess neurodegenerative diseases. The 2000s saw a continuous improvement in MRI image quality. Along with the development of 3D T1-weighted imaging (T1WI) with a spatial resolution of 1-2mm iso voxel, the volumetric measurement of brain structures, such as the hippocampus, advanced from manual region of interest methods to automated measurements using neuroimaging software like FreeSurfer and FMRIB Software library (FSL) (1, 2). The technique of voxel-based morphometry using Statistical Parametric Mapping (SPM) (3), which evaluates brain structures on a voxel-by-voxel basis, also progressed the field further. Additionally, the advent of functional MRI allowed for real-time visualization of active brain regions. The development of diffusion tensor imaging not only visualized white matter tracts but also enabled their quantitative analysis. These technologies confirmed organic brain changes in mood disorders, including major depressive disorder and Bipolar disorder.

Currently, MRI and neuroimaging techniques continue to evolve. With the latest MRI models, ultra-high-resolution imaging of less than 1mm, specifically 7T MRI, marks a transformative era in precision psychiatry, thanks to its unparalleled spatial resolution and signal-to-noise ratio. This leap in technology allows for individualized examinations, capturing the nuances of single participants’ brain structures and activities with great detail​. The advanced capabilities of 7T MRI in detecting subtle neurobiological changes are guiding research towards individualized psychiatric treatment plans, based on the unique brain structure or connectivity patterns of individuals (4). A manuscript in this Research Topic, “*Stress-related reduction of hippocampal subfield volumes in major depressive disorder: A 7-Tesla study*”, demonstrates that specific hippocampal subfield volumes, such as CA2/3, were reduced in people with major depressive disorder (MDD) compared to controls, and reductions in other subfields correlated with lifetime stressors (Alper et al.). The changes in specific hippocampal subfields may reflect underlying neurobiological characteristics of MDD, offering potential biomarkers for its diagnosis and for assessing treatment resistance.

While 7T MRI marks a technological advancement in MRI capabilities, the evolution of analysis techniques in 3T MRI systems is similarly advancing our ability to conduct detailed regional brain analyses (5). The features also include analyses dividing the cingulate gyrus and thalamus into subregions (6, 7). The study titled “*Amygdala’s T1-weighted image radiomics outperforms volume for differentiation of anxiety disorder and its subtype*” utilizes radiomics analysis to extract image features like texture and shape, aiming to detect subtle differences in amygdala impairments among anxiety disorder subtypes (Li et al.). Moreover, recent advances in cerebral cortical analysis introduced metrics (8), such as the Gyrification index and fractal coefficient, to evaluate the complexity of cortical structures, one example being the study “*Gyrification patterns in first-episode, drug-naïve major depression: Associations with plasma levels of brain-derived neurotrophic factor and psychiatric symptoms*.” (Natsuyama et al.) In addition to elucidating brain impairments in mood disorders, these new neuroimaging techniques are also expected to quantify therapeutic effects and provide deeper insights into the mechanisms of action of treatments. In “*The efficacy and cerebral mechanism of intradermal acupuncture for major depressive disorder: a study protocol for a randomized controlled trial*,” the protocol for a randomized controlled trial is described (Wu et al.). Utilizing the developed neuroimaging techniques, future research aims to validate the efficacy of intradermal acupuncture for major depressive disorder and elucidate its cerebral mechanism of action.

Resting-state functional MRI (fMRI) has also seen significant progress. The discovery of the three major networks - default mode network, salience network, and central executive network - in resting-state fMRI can be considered one of the most crucial advances in neuroscience since the 2000s (9). Initial analyses of Resting state functional MRI were based on the premise that connectivity during rest was constant, but recent research delves into the dynamic changes in connectivity (10). The study “*Abnormal dynamic functional network connectivity in people with early-onset bipolar disorder*” investigates the temporal changes in resting connectivity in early-onset bipolar disorder (Hu et al.). The authors reported people with early-onset BD had abnormal dynamic properties of brain functional network connectivity, which is indicative of unstable functional brain network connectivity. This was demonstrated through impaired coordination between cognitive and perceptual networks. Task-based fMRI has evolved, with a surge in studies using diverse cognitive tasks and emotional challenges.

In addition to MRI, there have been advancements in Electroencephalography (EEG) characterized by an increase in electrode count leading to higher resolution, as well as progress in cognitive tasks and emotional assignments. The study titled “*Electrophysiological Evidence for the Characteristics of Implicit Self-Schema and Other-Schema in Patients with Major Depressive Disorder: An Event-Related Potential Study*” investigated the neural correlates of self- and other-schemas in people with MDD (Yao et al.). The findings suggest that individuals with MDD exhibit distinct neural patterns, potentially reflecting a lack of positive self- and other-schemas and provide insights into the neural mechanisms underlying MDD, highlighting the importance of considering both self- and other-schemas in understanding and treating the disorder. Moreover, the research “*Face-specific negative bias of aesthetic perception in depression: behavioral and EEG evidence*” delved into the bias in aesthetic judgments in individuals with depression (Chen et al.). The aesthetic evaluation of unattractive faces was associated with decreased N200 negativity in the depression compared to controls, whereas the evaluation of beautiful faces was linked with decreased brain synchronization at the theta band. These findings are important for suggested design and development of aesthetics-oriented schemes in assisting the clinical diagnosis and therapy of MDD.

The objective of our present topic theme, which focuses on recent developments in neuroimaging in mood disorders, is to gain deeper insights into the cerebral alterations in mood disorders using newly proposed neuroimaging techniques. The research in this Research Topic enhances our understanding of brain changes in mood disorders, potentially guiding future efforts to identify early biomarkers, understand the natural progression of mood disorders, and develop targeted interventions from the outset of diagnosis. Further, this Research Topic includes several studies on people with first-episode and drug-naïve MDD, highlighting the pivotal role of neuroimaging in this nascent and critical area of research. As neuroimaging technology advances, the importance of investigating first-episode and drug-naïve MDD becomes increasingly apparent, providing unique insights into the neurobiological underpinnings of the disorder before the potential confounding effects of treatment.

## Author contributions

KW: Writing – original draft, Writing – review & editing. JJ: Writing – review & editing. RY: Writing – review & editing.

## References

[1] Jenkinson M Beckmann CF Behrens TE Woolrich MW Smith SM. FSL. Neuroimage. (2012) 62:782–90. doi: 10.1016/j.neuroimage.2011.09.015 21979382

[2] Fischl B. FreeSurfer. Neuroimage. (2012) 62:774–81. doi: 10.1016/j.neuroimage.2012.01.021 PMC3685476 22248573

[3] Ashburner J. SPM: a history. Neuroimage. (2012) 62:791–800. doi: 10.1016/j.neuroimage.2011.10.025 22023741 PMC3480642

[4] Neuner I Veselinović T Ramkiran S Rajkumar R Schnellbaecher GJ Shah NJ. 7T ultra-high-field neuroimaging for mental health: an emerging tool for precision psychiatry? Trans Psychiatry. (2022) 12:36. doi: 10.1038/s41398-022-01787-3 PMC8791951 35082273

[5] Iglesias JE Augustinack JC Nguyen K Player CM Player A Wright M. A computational atlas of the hippocampal formation using ex vivo, ultra-high resolution MRI: Application to adaptive segmentation of *in vivo* MRI. Neuroimage. (2015) 115:117–37. doi: 10.1016/j.neuroimage.2015.04.042 PMC4461537 25936807

[6] Saygin ZM Kliemann D Iglesias JE van der Kouwe AJW Boyd E Reuter M. High-resolution magnetic resonance imaging reveals nuclei of the human amygdala: manual segmentation to automatic atlas. Neuroimage. (2017) 155:370–82. doi: 10.1016/j.neuroimage.2017.04.046 PMC5557007 28479476

[7] Iglesias JE Insausti R Lerma-Usabiaga G Bocchetta M Van Leemput K Greve DN. A probabilistic atlas of the human thalamic nuclei combining ex vivo MRI and histology. Neuroimage. (2018) 183:314–26. doi: 10.1016/j.neuroimage.2018.08.012 PMC6215335 30121337

[8] Ruiz de Miras J Costumero V Belloch V Escudero J Ávila C Sepulcre J. Complexity analysis of cortical surface detects changes in future Alzheimer’s disease converters. Hum Brain Mapp. (2017) 38:5905–18. doi: 10.1002/hbm.23773 PMC5745046 28856799

[9] Menon V. Large-scale brain networks and psychopathology: a unifying triple network model. Trends Cognit Sci. (2011) 15:483–506. doi: 10.1016/j.tics.2011.08.003 21908230

[10] Chang C Glover GH. Time-frequency dynamics of resting-state brain connectivity measured with fMRI. Neuroimage. (2010) 50:81–98. doi: 10.1016/j.neuroimage.2009.12.011 20006716 PMC2827259

